# Tailoring luminescent oxygen sensitivity *via* structural design and its application in pressure-induced emission enhancement

**DOI:** 10.1039/d5sc05999b

**Published:** 2025-09-15

**Authors:** Hong-Jin Zhang, Zong-Ren Chen, Wan-Tao Chen, Jia-Wen Ye, Ling Chen

**Affiliations:** a Jiangmen Key Laboratory of Synthetic Chemistry and Cleaner Production, School of Environmental and Chemical Engineering, Wuyi University Jiangmen Guangdong 529000 PR China wyuchemyjw@126.com wyuchemcling@126.com; b School of Emergent Soft Matter, Center for Electron Microscopy, South China University of Technology Guangzhou 510006 China; c Guangdong Provincial Laboratory of Chemistry and Fine Chemical Engineering Jieyang Center Jieyang 515200 PR China

## Abstract

Pressure-induced emission enhancement (PIEE) is relatively rare and holds greater research promise than pressure-induced emission quenching. However, reports on PIEE often overlook the influence of atmospheric oxygen, especially in porous materials. Additionally, since the impact of energy transfer between the excited state of luminescent probes and T_1_(O_2_) on oxygen sensitivity remains unclear, tuning the luminescence-based oxygen sensitivity at the molecular structural level is highly challenging. Here, we report a series of coordination polymers (CuXBP, X = I, Br, Cl). Although they have similar structures and comparable porosity, their oxygen quenching efficiencies differ significantly (ranging from 95.8% to 7.7%). Computational simulations reveal that the superior oxygen quenching efficiency of CuIBP stems from the minimal Δ*E* (742 cm^−1^) between its T_1_ state and T_1_(O_2_). Additionally, under pressure, both CuClBP and CuBrBP exhibit the commonly observed red shift accompanied by luminescence quenching. However, CuIBP displays the less commonly observed PIEE, attributed to the reduced porosity after grinding, which lowers oxygen-sensing efficiency (from 95.8% to 33.2%). Compared to its unground state, this efficiency reduction in CuIBP substantially attenuates oxygen-induced quenching, resulting in stronger luminescence (>2-fold enhancement) under ambient conditions. This work establishes a novel strategy for designing oxygen sensing and PIEE materials.

## Abstract 2

Pressure-induced emission enhancement (PIEE) is achieved *via* tuning oxygen-sensing sensitivity.

## Graphical abstract

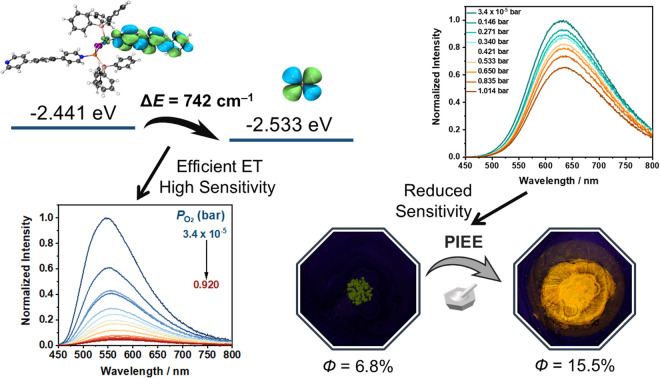

## Introduction

The investigation of luminescent materials responsive to environmental stimuli remains a key research focus.^1–3^ Materials exhibiting unique luminescence changes under stimuli such as light,^4^ heat,^5^ pressure^6^ and gases^7^ demonstrate promising applications in sensors, optical switches, and so on.^8–10^ Oxygen, as a gas of high monitoring significance in environmental surveillance, biomedicine, and industrial production, has driven the development of materials with high-efficiency oxygen-sensing capabilities.^11–13^ Precise modulation of oxygen sensitivity remains a highly significant topic in the development of luminescent oxygen-sensing materials.^14,15^ Common strategies such as enlarging material porosity or diluting optical probe concentration effectively boost sensitivities.^16–18^ However, the engineering of molecular photophysical properties to control oxygen sensitivity has not been systematically explored, because how energy transfer between the excited state of luminescent probes and the excited state of oxygen impacts oxygen sensitivity remains unclear.^19^

Pressure-induced luminescence variation usually manifests as red shifts in wavelengths and quenching of intensity,^20^ originating from molecular structure planarization,^21^ shortened metal–metal distances,^22^ increased packing density,^23^ and altered molecular interactions.^24^ Notably, recent reports have increasingly emerged on suppressed blue shifts or enhancement in luminescence,^25–27^ which provide novel insights for developing advanced pressure-responsive materials. For instance, introducing dynamic hydrogen-bonding networks or halogen-bonding interactions into rigid conjugated frameworks can counteract energy dissipation from molecular vibrations or suppress excessive H-aggregation quenching effects.^28–30^ Furthermore, aggregation-induced emission luminogens (AIEgens) offer unique advantages, as pressure-driven molecular aggregation effectively restricts intramolecular rotation (RIR), enabling luminescence enhancement.^31–33^ These designs transcend the traditional paradigm of pressure-induced emission quenching (PIEQ). However, nearly all pressure application operations are conducted in air. The influence of oxygen on the luminescence of materials before and after compression should be considered, especially for porous materials, but relevant studies have not yet been reported.

Cu(i) clusters have gradually garnered increasing attention for their oxygen-quenching or pressure-induced luminescence responses, owing to their rich transition modes, such as metal-to-ligand charge transfer (MLCT), halogen-to-ligand charge transfer (XLCT), cluster-centered (CC) transitions and Cu(i)⋯Cu(i) interactions, which exhibit high sensitivity to environmental stimuli.^33–36^

Here, we report a series of Cu(i) cluster-based coordination polymers (CPs), denoted as CuXBP (X = I, Br, Cl). Although they have similar structures and comparable porosity, their oxygen quenching efficiencies differ significantly. To be specific, in 1 bar O_2_, emission intensities are quenched by 95.8%, 62.5%, and 7.7% for CuIBP, CuBrBP and CuClBP, respectively. Computational studies reveal that the energy gaps between their triplet excited states (T_1_) and the triplet excited state energy level of O_2_ (T_1_(O_2_)) govern the sensitivities in luminescent oxygen sensing. Among them, the LUMO of CuIBP is closest to T_1_(O_2_) in energy, resulting in its highest oxygen sensing efficiency. In addition, grinding reduces oxygen-sensing efficiencies across the series, with CuIBP exhibiting the most severe attenuation (from 95.8% to 33.2%). This efficiency loss leads to a >2-fold enhancement of its photoluminescence quantum yield (*Φ*) under ambient conditions, demonstrating pressure-induced emission enhancement (PIEE). However, CuBrBP and CuClBP display normal PIEQ under pressure, because their oxygen sensitivity undergoes minimal changes before and after grinding. This work is the first to systematically investigate the connection between oxygen sensing and PIEE, proposing a novel strategy for designing PIEE materials through the regulation of oxygen sensing properties.

## Results and discussion

**Fig. 1:**
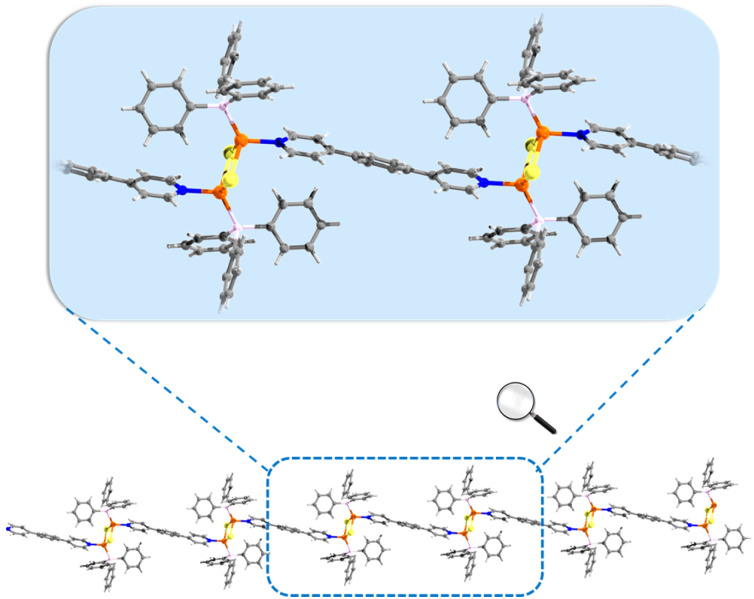
Chain structure of CuIBP. Colour code: Cu, orange; N, blue; I, yellow; P, pink; C, grey; H, light grey.

A series of Cu(i) CPs formulated as [Cu_2_X_2_(TPP)_2_(BPB)]·g (X = I, Br, Cl; TPP = triphenylphosphine; BPB = 1,4-bis(pyridin-4-yl)benzene; g = methanol) and denoted as CuXBP·M, were synthesized through the reaction of CuX with TPP and BPB ligands (Scheme. S1 and Table S1). Through heating, the guest-free CuXBPs can be obtained. Single-crystal X-ray diffraction (SCXRD) reveals the absence of methanol guest molecules in the CuXBP structure. CuXBPs crystallize in the triclinic *P*-1 space group, featuring an asymmetric unit containing a rhombic [Cu_2_X_2_] cluster coordinated by one TPP ligand and half of a BPB ligand (Table S2 and Fig. S1). Notably, systematic expansion of their unit cell volumes correlates with the increase in the halide ionic radius (I^−^ > Br^−^ > Cl^−^, Table S2). The [Cu_2_X_2_] clusters undergo directional extension *via* BPB linkers to generate molecular chains and are capped by TPP ligands (Fig. 1 and S2). Interchain π–π interactions between aromatic moieties facilitate the assembly of these linear arrays into 3D supramolecular architectures (Fig. S3). In the structures of CuXBPs, all Cu–Cu distances exceed 3 Å (CuIBP: 3.18 Å; CuBrBP: 3.08 Å; CuClBP: 3.03 Å), indicating negligible Cu⋯Cu interactions in these CPs (Table S3). The phase purities of CuXBP were verified by powder X-ray diffraction (PXRD, Fig. S4–S6), and the elemental analysis results are listed in Table S4. Thermogravimetric analysis (TGA) further confirms that there are no guest molecules in CuXBP (Fig. S7). Remarkably, the stability of CuXBP is maintained after 12 months under ambient storage conditions (Fig. S4–S6).

The solid-state ultraviolet-visible (UV-vis) absorption spectra of CuIBP, CuBrBP, and CuClBP exhibit similar broad bands at 200–500 nm (Fig. S8), consistent with their yellow colour. Density functional theory (DFT) and time-dependent density functional theory (TDDFT) calculations of S_0_ → S_1_ transitions in CuXBP confirm that these absorptions arise from mixed MLCT and XLCT (M/XLCT) transitions (Fig. S9). Notably, the XLCT contribution progressively intensifies across the CuXBP series (Cl^−^ < Br^−^ < I^−^), as quantified by the orbital composition analyses in Table S5. Under 365 nm excitation, the maximum emission wavelength (*λ*_em_) of CuXBPs is located at 542 nm, 577 nm, and 605 nm for CuIBP, CuBrBP, and CuClBP, respectively, demonstrating a systematic red shift progressing from I^−^ to Br^−^ to Cl^−^ (Fig. S10). TDDFT calculations also attribute this red-shift trend to the increasing MLCT : XLCT ratio in S_1_ → S_0_ transitions (I^−^ < Br^−^ < Cl^−^, Table S6).

**Fig. 2:**
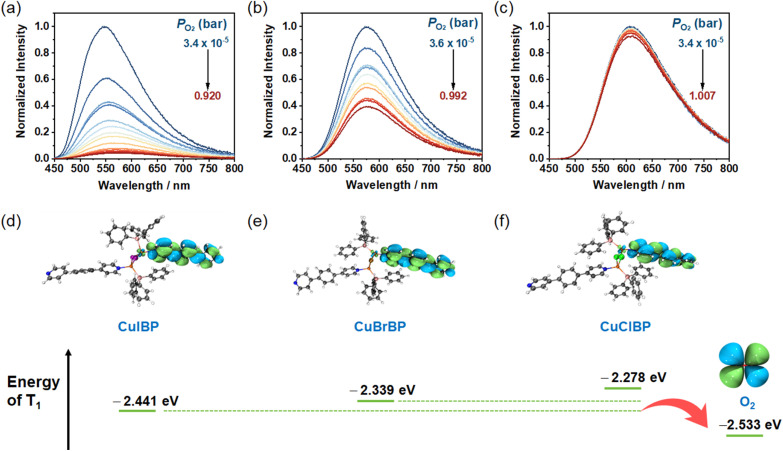
Emission spectra of (a) CuIBP, (b) CuBrBP and (c) CuClBP at different O_2_ pressures, excited at 365 nm. (d–f) The triplet excited state of CuXBPs (X = I, Br, Cl) and O_2_, evaluated by TDDFT calculations.

CuXBPs exhibit oxygen-sensitive luminescence due to quasi-discrete pores in their structures, which allow O_2_ permeation and facilitate dynamic collisions between O_2_ and the emissive centers. The calculated voids are 6.5%, 6.3%, and 4.8% for CuIBP, CuBrBP, and CuClBP, respectively (Fig. S11). To be specific, for CuIBP, the emission intensity progressively decreases with increasing oxygen pressure, and CuIBP exhibits 95.8% quenching efficiency in 1 bar O_2_ (Fig. 2a). This efficiency is significantly higher than the values of 62.5% and 7.7% for CuBrBP (Fig. 2b) and CuClBP (Fig. 2c), respectively. Sensing reversibility and response kinetics were investigated through *in situ* emission monitoring under alternating vacuum and O_2_ conditions (Fig. S12–S14). Remarkably, all CuXBPs maintain >95% initial intensity after continuous UV irradiation for 10^4^ seconds, demonstrating exceptional photostability (Fig. S15). Moreover, linear Stern–Volmer analysis (*I*_0_/*I* = 1 + *K*_SV_*P*_O2_) reveals dynamic quenching behaviour, and the calculated *K*_SV_ (Stern–Volmer quenching constant) values decrease sequentially from 22.95 per bar to 1.65 per bar and 0.08 per bar for CuIBP, CuBrBP and CuClBP, respectively (Fig. S16). Even though all three complexes show similar structures and comparable porosity, their sensitivity to oxygen differs by dozens of times.

Common methods for regulating oxygen quenching efficiency, such as adjusting porosity or dispersing probe molecules, fail to adequately explain the significant differences in oxygen sensitivity observed among the CuXBPs in this system. Luminescent oxygen sensing is generally understood to occur *via* energy transfer between the luminescent probe and oxygen. Such energy transfer requires relative matching of energy levels between excited states, analogous to the energy transfer mechanism between the lanthanide and ligands in lanthanide complexes.^37^ Inspired by this principle and supported by our observation of varying emission peak energies (differing *λ*_em_) among the CuXBPs, we hypothesize that differences in the T_1_ excited state energy of the complexes constitute the paramount factor responsible for their distinct oxygen sensitivities. Therefore, we investigated the T_1_ energies of CuXBPs using TDDFT calculations. These calculations indicate gradual T_1_ (lowest unoccupied molecular orbital, LUMO) energy elevation across the CuXBPs, specifically –2.441, –2.339, and –2.278 eV for CuIBP, CuBrBP and CuClBP, respectively, with all LUMO levels exceeding the T_1_(O_2_) state (–2.533 eV). This demonstrates that the energy gap between the LUMO of CuIBP and the T_1_(O_2_) state is the smallest (0.092 eV, corresponding to 742 cm^−1^), which consequently enables the most effective energy transfer between their excited states (Fig. 2d–f), leading to a *K*_SV_ of 22.95 per bar. Meanwhile, the energy gaps between the LUMOs of CuBrBP and CuClBP and the T_1_(O_2_) state are 1565 and 2057 cm^−1^, respectively. These larger energy gaps significantly weaken the energy transfer efficiency and therefore result in reduced *K*_sv_ values of 1.65 per bar and 0.08 per bar for CuBrBP and CuClBP, respectively. Moreover, CuIBP shows a drastic reduction from 13.34 μs (vacuum) to 0.38 μs (O_2_), while CuBrBP (6.01 → 2.18 μs) and CuClBP (1.42 → 1.35 μs) exhibit more modest changes (Fig. S17–S19).

To investigate the influence of pressure on the oxygen sensing performance of the CuXBPs, we subjected the CuXBPs to grinding treatment, and the resulting samples were named CuXBP-G (X = I, Br, Cl). The ground powders exhibit broad amorphous PXRD patterns (Fig. S20–S22), indicating disruption of the crystal packing. Fourier transform infrared spectroscopy (FT-IR) analysis confirms the absence of chemical bond cleavage or formation during mechanical treatment, as evidenced by unchanged vibrational peaks in both CuXBP and CuXBP-G (Fig. S23–S25), suggesting that mechano-induced property modifications primarily arise from structural reorganization rather than covalent bond alterations.

**Fig. 3:**
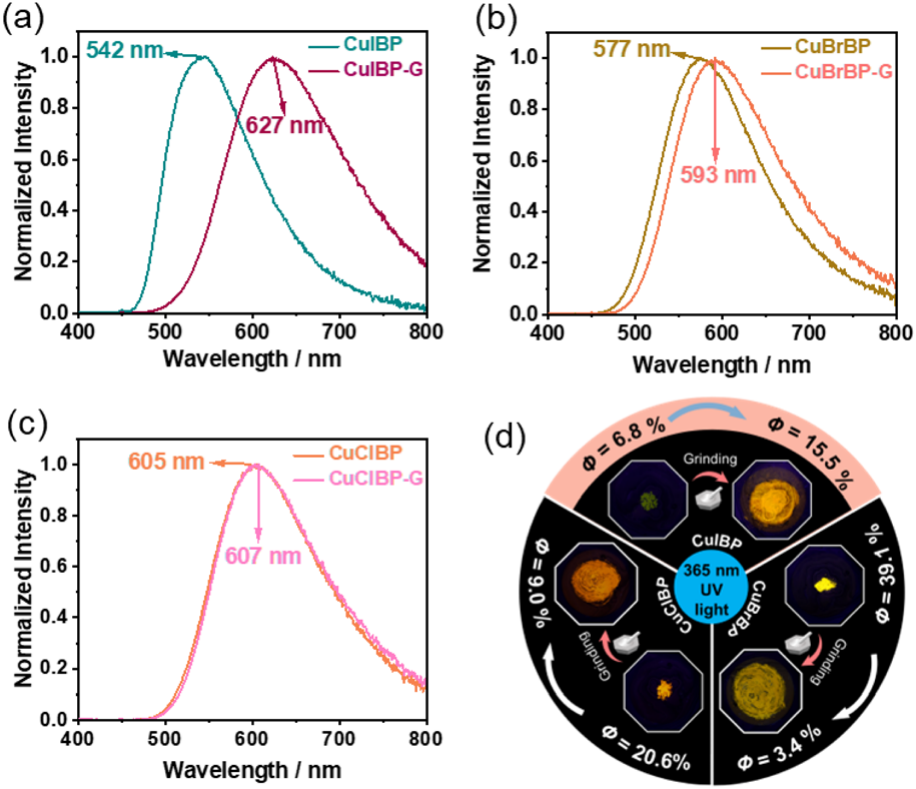
Emission spectra of (a) CuIBP, (b) CuBrBP and (c) CuClBP before and after grinding, with an excitation wavelength of 365 nm. (d) Photographs and *Φ* of CuXBPs (X = I, Br, Cl) under 365 nm UV light before and after grinding.

As shown in Fig. 3, CuIBP-G displays orange-yellow luminescence with *λ*_em_ at 627 nm, showing a red shift of 85 nm compared to that of CuIBP (542 nm). CuBrBP-G (593 nm) and CuClBP-G (607 nm) show 16 nm and 2 nm red shifts compared to CuBrBP (577 nm) and CuClBP (605 nm), respectively. These red shifts in the emission spectra are likely attributed to the planarization of the ligands in the complexes induced by grinding, as well as enhanced intermolecular interactions between the ligands. The UV-Vis spectra of CuXBP-G exhibit broadened absorption ranges (200–550 nm), with a slight red shift compared to those of CuXBP (Fig. S26–S28).

Mechanical grinding induces denser particle packing under pressure, significantly reducing oxygen permeability through constricted pore channels and consequently diminishing oxygen quenching efficiency. As demonstrated in Fig. S29a, CuIBP-G exhibits 33.2% luminescence quenching in 1 bar O_2_, representing only 1/3 of the quenching efficiency of CuIBP. Nevertheless, it maintains excellent linear Stern–Volmer quenching behaviour across varying oxygen partial pressures, and the *K*_SV_ value was calculated to be 0.51 per bar (Fig. S29d). The linear Stern–Volmer plot indicates that the grinding is even and thorough. If both ground and unground samples were present, the SV curve would bend and yield two *K*_SV_ values. In contrast, CuBrBP-G shows 28.3% quenching efficiency (Fig. S29b) with *K*_SV_ = 0.39 per bar (Fig. S29e), while CuClBP-G displays minimal quenching at 5.3% (Fig. S29c) and a substantially reduced *K*_SV_ of 0.055 per bar (Fig. S29f). Though CuXBPs exhibit decreases in oxygen response capability after grinding, they still show good photostable behaviour (Fig. S30).

Notably, after grinding, *Φ* of CuIBP shows a significant enhancement (from 6.8% to 15.5%), whereas those of CuBrBP and CuClBP decrease from 39.1% to 3.4% and 20.6% to 9.0%, respectively (Fig. 3d). As shown in the photographs of Fig. 3d, CuIBP can be observed to have a greatly enhanced emission after grinding.

**Fig. 4:**
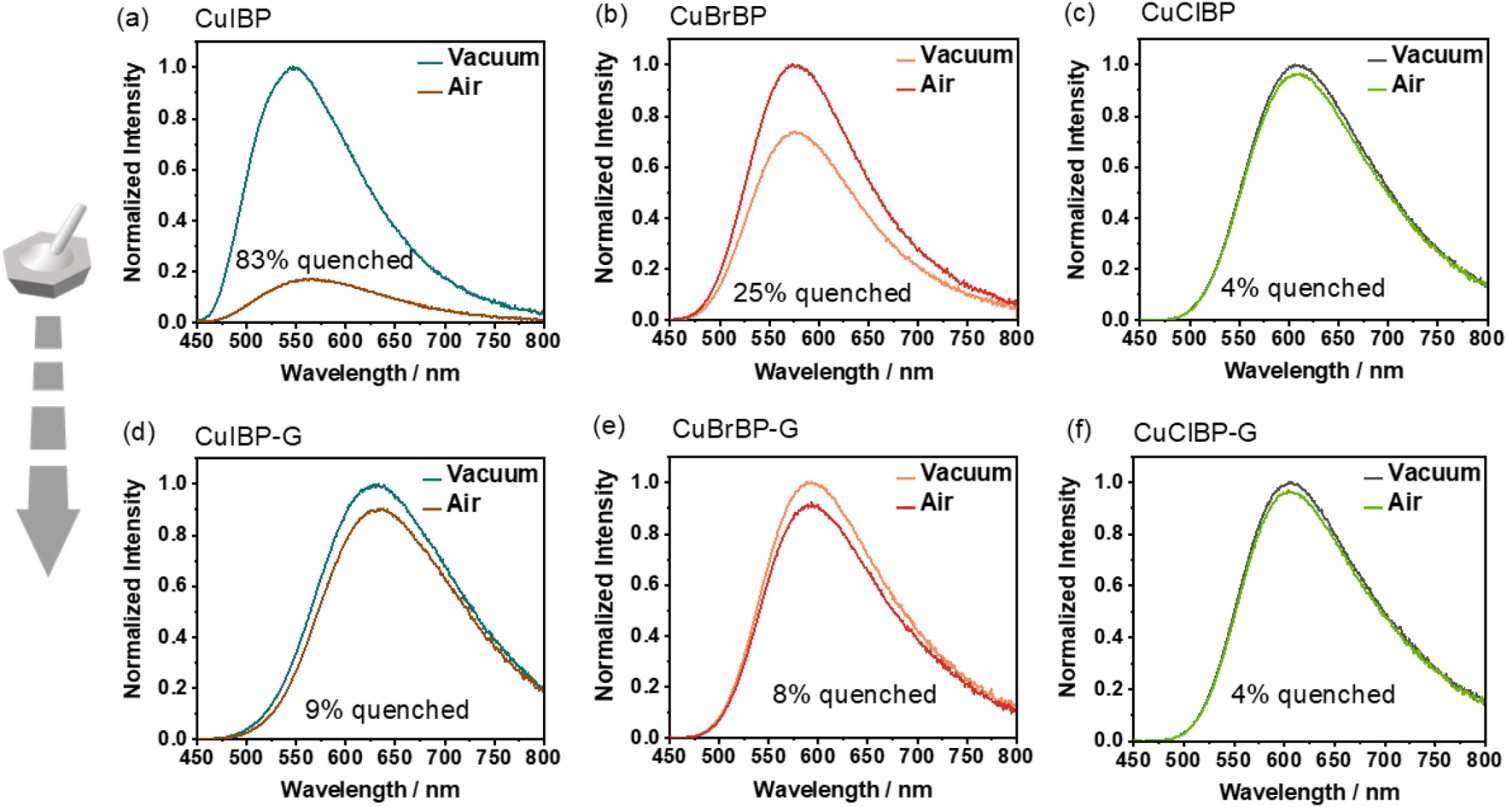
The emission changes of CuXBPs (X = I, Br, Cl) in vacuum and air (a–c) before and (d–f) after grinding.

To further explain the differing trends in *Φ* of CuXBPs in air before and after grinding, we analysed and compared the changes in the luminescence emission intensity of these CuXBPs in vacuum and air before and after grinding. As shown in Fig. 4a and d, before grinding, the luminescence of CuIBP in air was significantly quenched (83%), whereas after grinding, the quenching effect of air was greatly weakened (9%), thus demonstrating PIEE under ambient conditions. In contrast, CuBrBP (25–8%) and CuClBP (4–4%) show a negligible change in the luminescence quenching effect by air before and after grinding (Fig. 4b, c, e and f). Therefore, the observed overall luminescence weakening for CuBrBP and CuClBP after grinding should be primarily attributed to enhanced non-radiative transitions, consistent with established pressure effects reported in most literature. These observations suggest a viable strategy: precise regulation of oxygen sensing sensitivity could enable tailored control over pressure-induced luminescence enhancement/quenching characteristics. The photophysical properties for CuXBP before and after grinding are summarized in Table 1. In addition, the luminescence lifetimes of the ground samples measured in air, 1 bar O_2_, and vacuum are shown in Fig. S31–S33.

**Table 1:** Summary table of the photophysical properties of CuXBP

Compound	*λ*_em_ a/nm	*Φ* a/%	Quenched Emission^b^/%	*K*_SV_/bar^−1^
CuIBP	542	6.8	**83**	**22.95**
CuIBP-G	627	15.5	9	0.51
CuBrBP	577	39.1	**25**	**1.65**
CuBrBP-G	593	3.4	8	0.39
CuClBP	605	20.6	**4**	**0.08**
CuClBP-G	607	9.0	4	0.055

a Data in air.

b Quenched emission from vacuum to air.

## Conclusions

We successfully synthesized three Cu(i) CPs that have similar structures and comparable porosity, but exhibit distinct O_2_-induced luminescence quenching efficiencies and pressure-dependent emissive behaviors. Based on these three complexes, we revealed the critical role of triplet energy alignment between the complexes and oxygen in facilitating energy transfer during dynamic luminescence quenching. This work introduces the concept of modulating oxygen sensing efficiency through regulation of complex triplet energy. Furthermore, by investigating differential oxygen quenching effects before and after grinding in air, we established that the PIEE observed in one complex originates from “pressure-attenuated oxygen quenching”. This led to the proposal for designing novel pressure sensing materials by leveraging environmental oxygen's impact on luminescence. Our findings provide valuable design principles for developing oxygen-sensing and pressure-sensing materials.

## Author contributions

Jia-Wen Ye designed the research. Hong-Jin Zhang performed all syntheses and most measurements. Zong-Ren Chen and Wan-Tao Chen offered help for crystallographic data analysis. Jia-Wen Ye and Ling Chen analysed data and wrote the manuscript.

## Conflicts of interest

The authors declare that they have no conflict of interest.

## Supplementary Material

SC-OLF-D5SC05999B-s001

SC-OLF-D5SC05999B-s002

## Data Availability

CCDC 2473355–2473360 contain the supplementary crystallographic data for this paper.^38^ All data are available in the main text and SI. Supplementary information: Materials and crystallographic and photoluminescence studies. See DOI: https://doi.org/10.1039/d5sc05999b.
