# Supplementary material for: Tailoring luminescent oxygen sensitivity via structural design and its application in pressure-induced emission enhancement
Source: Chem Sci. 2025 Sep 15;16(40):18799–805. doi: 10.1039/d5sc05999b (PMC12434614; doi:10.1039/d5sc05999b)
Supplement: SC-016-D5SC05999B-s001 [file SC-016-D5SC05999B-s001.pdf]

Supplementary Information (SI) for Chemical Science.
This journal is © The Royal Society of Chemistry 2025

Electronic Supplementary Information

Tailoring Luminescent Oxygen Sensitivity via Structural Design and Its Application in Pressure-Induced Emission Enhancement

Hong-Jin Zhang,[a] Zong-Ren Chen,[a] Wan-Tao Chen[b], Jia-Wen Ye*[ac] and Ling Chen*[ac]

[a] Jiangmen Key Laboratory of Synthetic Chemistry and Cleaner Production, School of Environmental and Chemical Engineering, Wuyi University, Jiangmen, Guangdong 529000, PR China; E-mail: wyuchemyjw@126.com; wyuchemcling@126.com

[b] School of Emergent Soft Matter, Center for Electron Microscopy, South China University of Technology, Guangzhou, 510006, China.

[c] Guangdong Provincial Laboratory of Chemistry and Fine Chemical Engineering Jieyang Center, Jieyang 515200, PR China.

Experimental Procedures

S1. Materials and Physical Measurements.

All commercially available reagents and solvents were used as received without further purification. Elemental analysis (EA) was recorded by a Vario EL cube elemental analyzer. Powder X-ray diffraction (PXRD) patterns were recorded by a Rigaku Mini powder X-ray diffractometer. Solid-state UV-vis absorption spectra were recorded on a PerkinElmer Lambda950 UV-vis-NIR absorption spectrometer coupled with an integrating sphere by using $BaSO_4$ powder as the reflectance reference. Thermogravimetric (TG) analyses were performed by a Netzsch TG209F1 Libra R instrument with a ramping rate of 10.0 °C min^{-1} under N_2 atmosphere. Fourier transform infrared spectroscopy (FT-IR) spectra were recorded on a PerkinElmer Frontier FTIR spectrometer.

Single-Crystal X-Ray Diffraction. Single crystal diffraction data were collected on a Rigaku XtaLAB single-crystal X-ray diffractometer by using Cu-Kα radiation (λ = 1.54184 Å). The structures were solved with the direct methods and refined with the full-matrix least-squares method on F^2 using the SHELXTL package. SHELXT was used for the structure solution of the crystals. All non-hydrogen atoms were refined with anisotropic displacement parameters, and hydrogen atoms were placed in the idealized positions and refined as rigid atoms with the relative isotropic displacement parameters. Additional crystallographic information is shown in Tables S1-S3.

Photoluminescence Measurement. The emission spectra were recorded by Edinburgh FLS1000 fluorescence spectrometer equipped with a continuous Xe lamp. All instrument parameters such as the excitation split, emission split and scanning speed were fixed during the *in-situ* measurements. Photoluminescence quantum yields (Φ) were performed by the same spectrometer in the integrating sphere. Luminescence lifetime experiments were performed by the Edinburgh FLS1000 fluorescence spectrometer equipped with a variable pulsed laser (VPL) at 375±10 nm as the excitation source.

Density Functional Theory (DFT) Calculations: DFT calculations were performed using Gaussian 09 program.[1] Geometry optimizations were performed using by PBE0 density function.[2] The 6-31G(d) basis set were used for C, N and H, while LanL2DZ pseudopotentials were used for Cl, P, Br, Cu and I. The unrestricted DFT calculations were

employed to optimize the T_1 structure with the same function and basis set.

Time-Dependent Density Functional Theory (TDDFT) Calculations. TDDFT calculations were used to investigate the photophysical properties of the compounds. The calculations were performed with the same function and basis set as described at the DFT optimizations. The first 15 singlet states without geometry optimization were calculated for the transition of $S_1 \rightarrow S_0$. The first 5 triplet states without geometry optimization were calculated for the transition of $T_1 \rightarrow S_0$ based on the T_1 structure, which is optimized by the unrestricted DFT calculation. The Milliken orbital composition analyses were calculated by the Multiwfn program.[3]

O_2 Sensing Property Measurement. The O_2 luminescence sensing properties were measured in situ by placing CuXBP (X = I, Br, Cl) inside an Oxford variable-temperature device with a four-way valve that connected the sample chamber to a pressure gage, a vacuum pump and a O_2 cylinder. The pressure of O_2 was recorded by Sensor PIZA 111. The emission spectra and the decay curves were recorded by Edinburgh FLS1000 luminescence spectrometer equipped with a continuous Xe lamp. All instrument parameters such as the excitation split, emission split and scanning speed were fixed during the in-situ measurements.

S2. Details of Synthesis.

Synthesis of CuIBP ($C_{52}H_{42}Cu_2I_2N_2P_2$) Single Crystals. BPB (1,4-bis(pyridin-4-yl)benzene, 47.0 mg, 0.2 mmol) and PPh_3 (triphenylphosphine, 106 mg, 0.4 mmol) were put into a 20 mL bottle. Then 10 mL of CH_3CN was added and ultrasonicated for 3 minutes. After adding CuI (77.0 mg, 0.4 mmol), the mixture was left for 24 h to get yellow crystals. The crystals were collected and washed with methanol 3 times a day for a week. Finally, dried at 60 ^{o}C and the yield based on CuIBP is 65-75%.

Synthesis of CuBrBP ($C_{52}H_{42}Cu_2Br_2N_2P_2$) Single Crystals. Similar to the method for synthesizing CuIBP crystals, the difference is that CuBr (57.0 mg, 0.4 mmol) is used instead of CuI. After removing the guest molecules, yellow crystals of CuBrBP are collected, and the yield based on CuBr is 65-75%.

Synthesis of CuClBP ($C_{52}H_{42}Cu_2Cl_2N_2P_2$) Single Crystals. Similar to the method for synthesizing CuIBP crystals, the difference is that CuCl (39.0 mg, 0.4 mmol) is used instead of CuI. After removing the guest molecules, yellow crystals of CuClBP are collected, and the yield based on CuClBP is 65-75%.

Synthesis of CuXBP (X = I, Br, Cl) Powder. The same method as synthesis of CuXBP (X = I, Br, Cl) single crystal was employed, expect stirring for a day to get the powder.

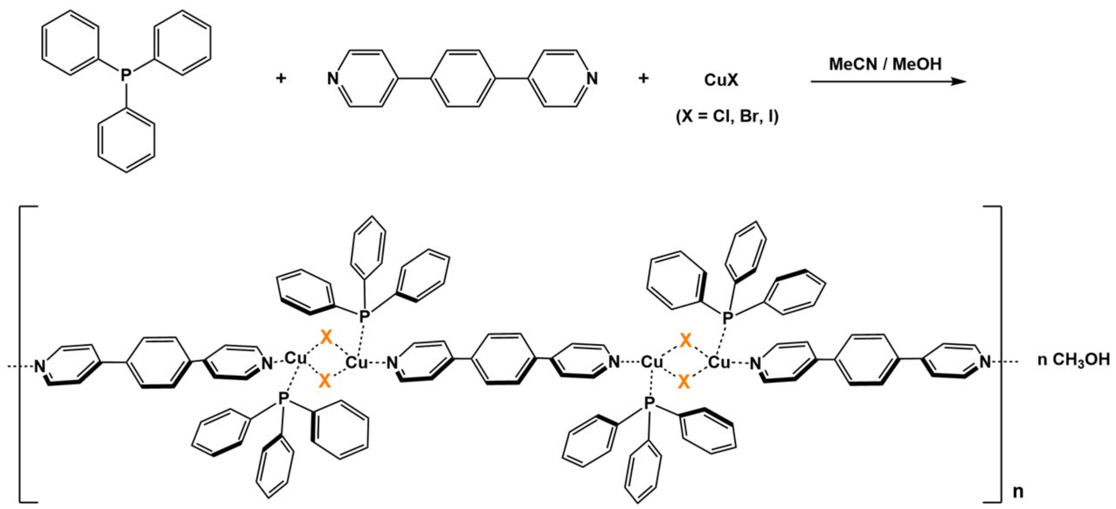

Scheme S1. Schematic diagram of the synthesis for CuXBP·CH_3OH (X = I, Br, Cl).

Figures and Tables

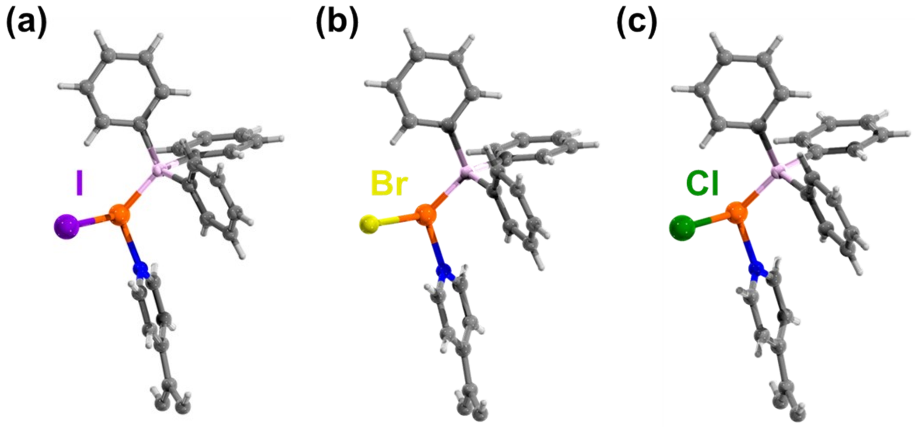

Figure S1. The asymmetric units of (a) CuIBP, (b) CuBrBP, (c) CuClBP, respectively. Colour codes: C, dark grey; H, light grey; N, blue; Cu, orange; P, pink; Cl, green; Br, yellow; I, purple.

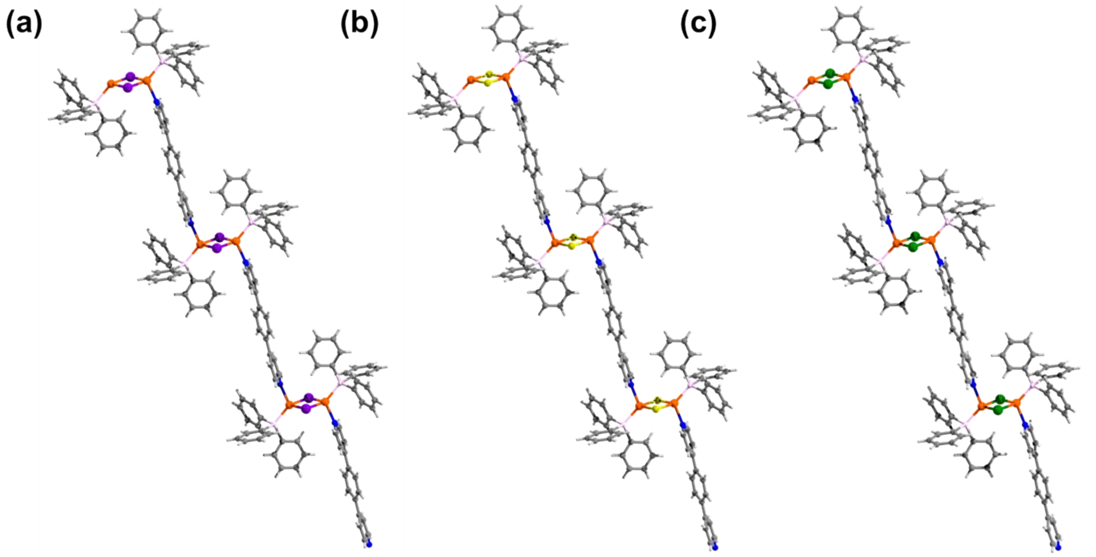

Figure S2. The chain structures of (a) CuIBP, (b) CuBrBP, (c) CuClBP, respectively. Colour codes: C, dark grey; H, light grey; N, blue; Cu, orange; P, pink; Cl, green; Br, yellow; I, purple.

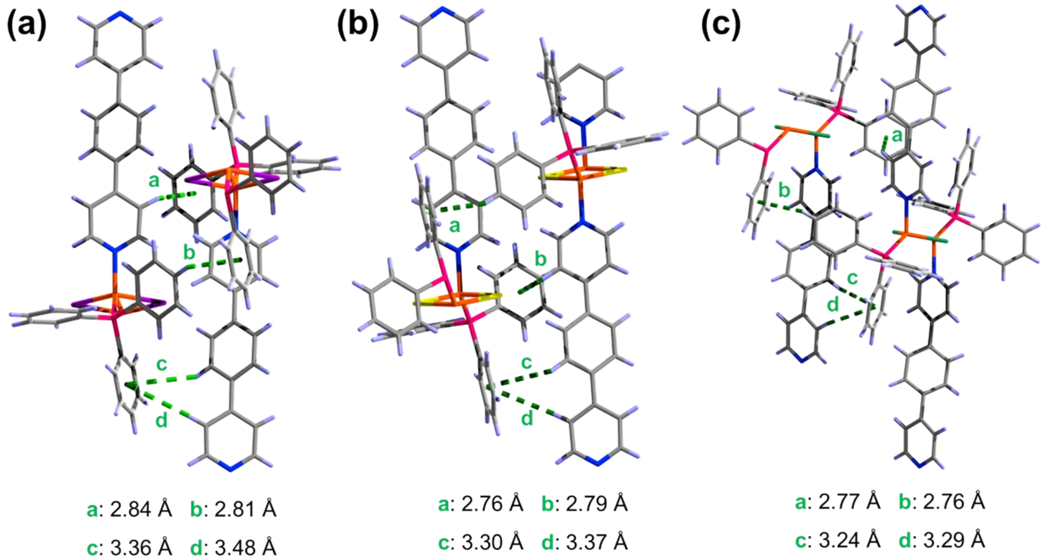

Figure S3. The molecular chains in (a) CuIBP, (b) CuBrBP, (c) CuClBP that are stacked by C-H···π interactions. Colour codes: C, grey; H, light purple; N, blue; Cu, orange; P, pink; Cl, green; Br, yellow; I, purple.

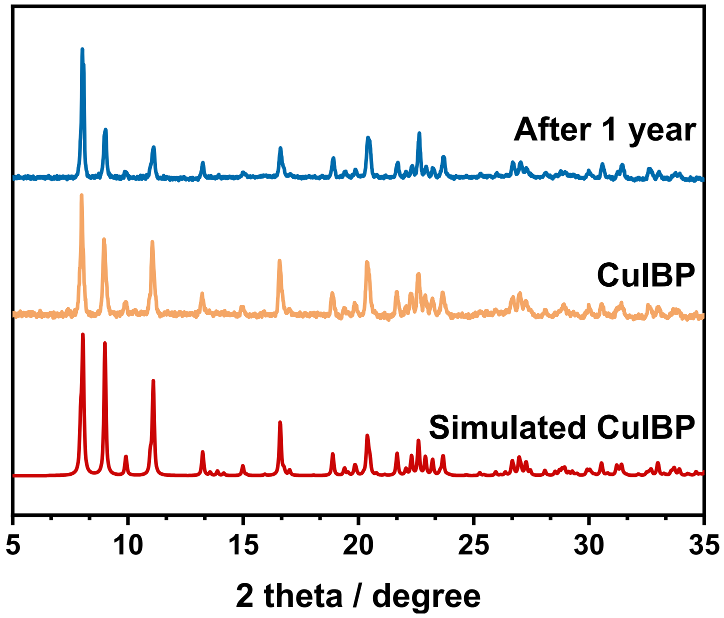

Figure S4. PXRD patterns of as-synthesized CuIBP and of the sample exposed to air for 1 year.

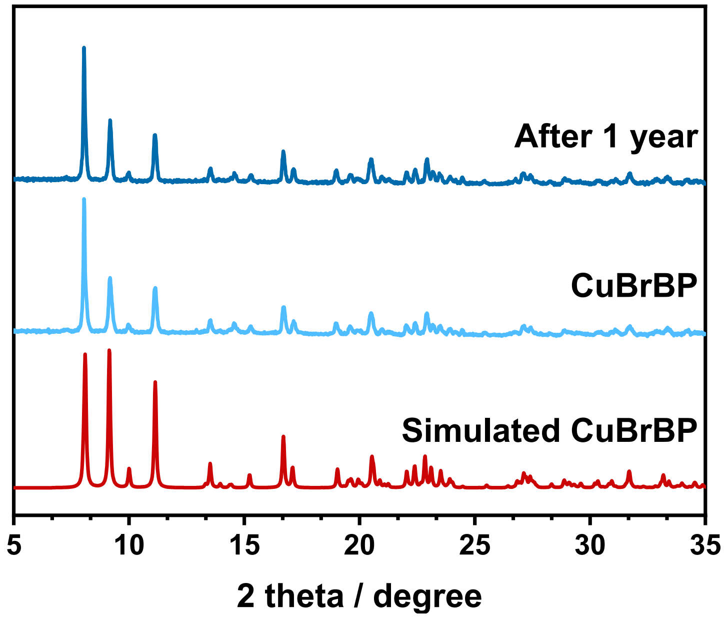

Figure S5. PXRD patterns of as-synthesized CuBrBP and of the sample exposed to air for 1 year.

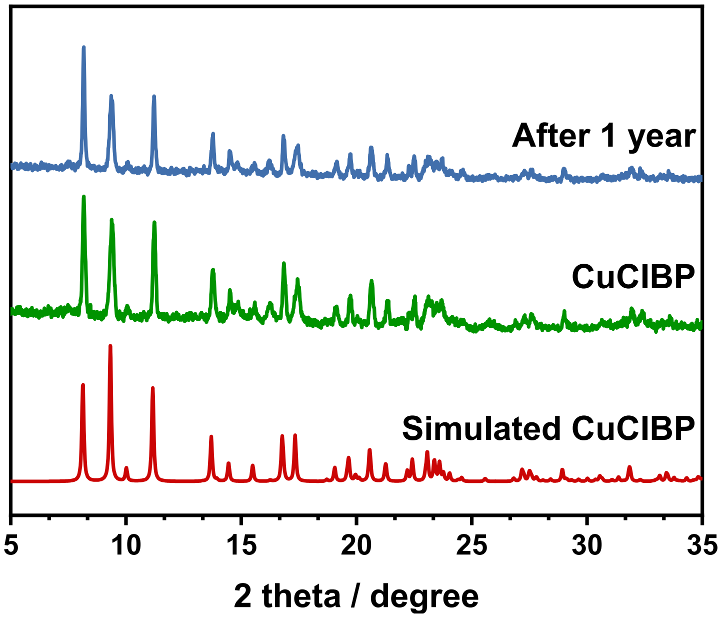

Figure S6. PXRD patterns of as-synthesized CuClBP and of the sample exposed to air for 1 year.

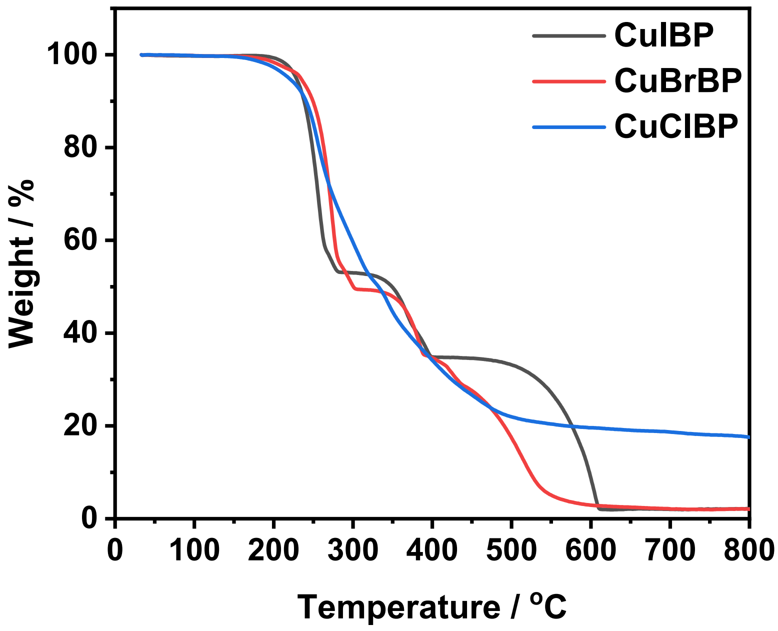

Figure S7. TG curves of CuXBPs.

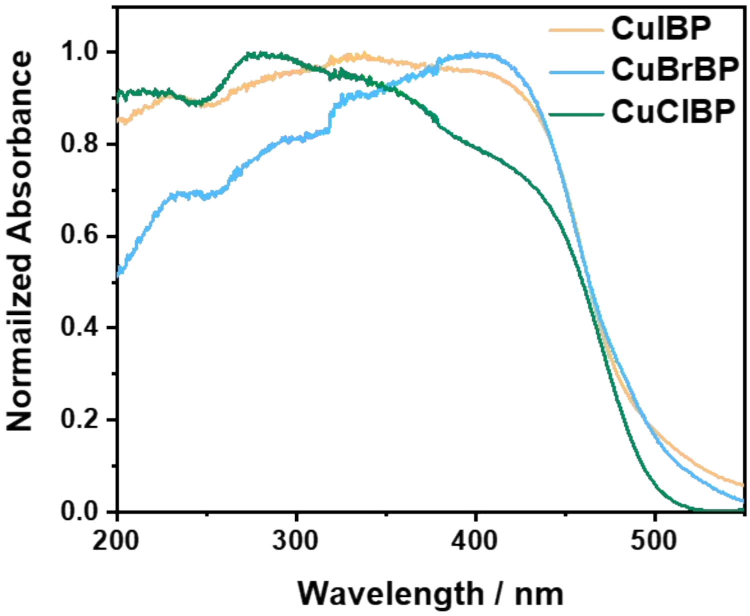

Figure S8. Solid-State UV-vis absorption spectra of CuXBPs.

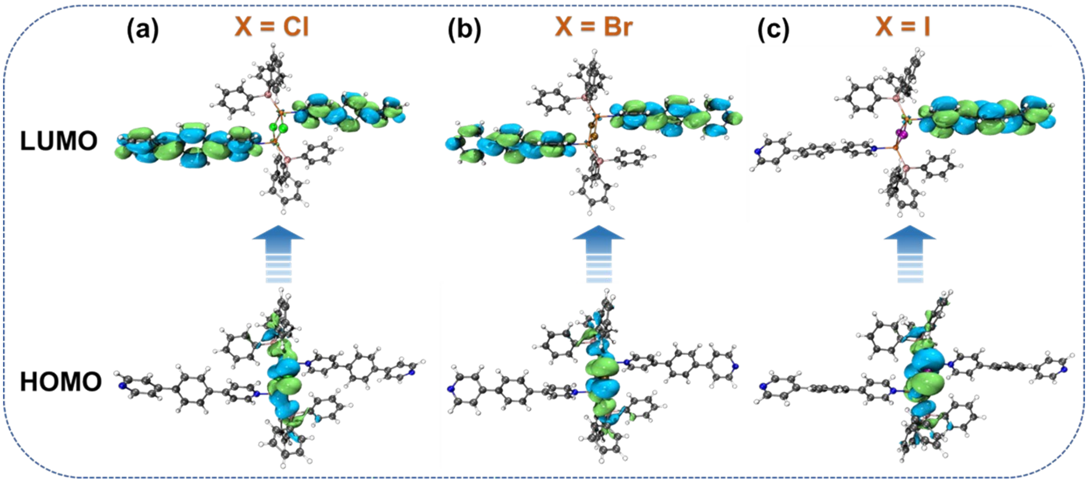

Figure S9. Molecular orbitals ($S_0 \rightarrow S_1$ transitions) of (a) CuClBP, (b) CuBrBP, (c) CuIBP, evaluated by TDDFT calculations.

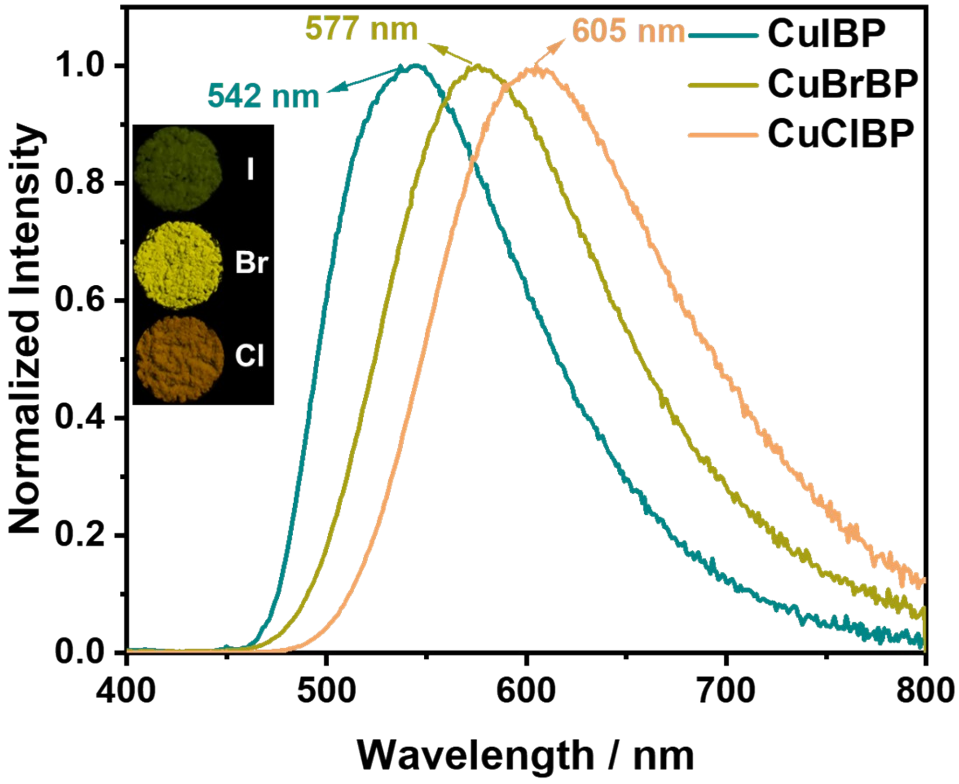

Figure S10. Emission spectra of CuXBPs, under 365 nm excitation at ambient conditions. Insert: the photographs of the CuXBPs under 365 nm excitation.

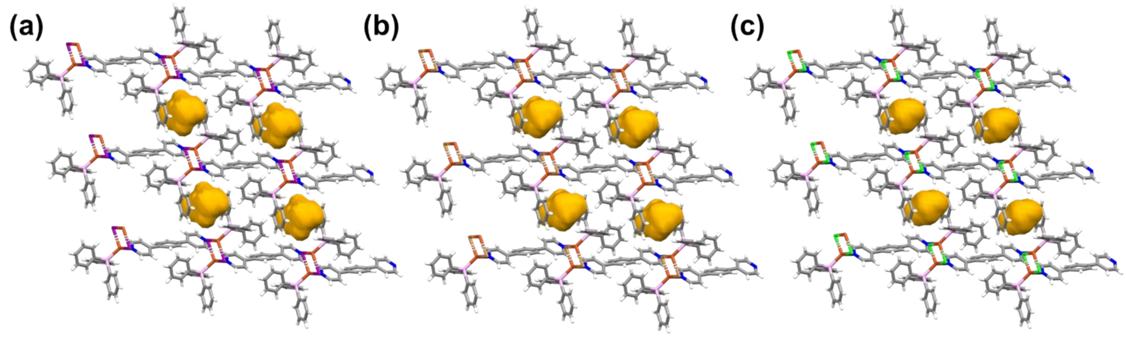

Figure S11. The pore surface observed from the perspective along the *a*-axis. (a) CuIBP, (b) CuBrBP, (c) CuClBP, respectively. Colour codes: C, grey; H, white; N, blue; Cu, orange; P, pink; Cl, green; Br, yellow; I, purple.

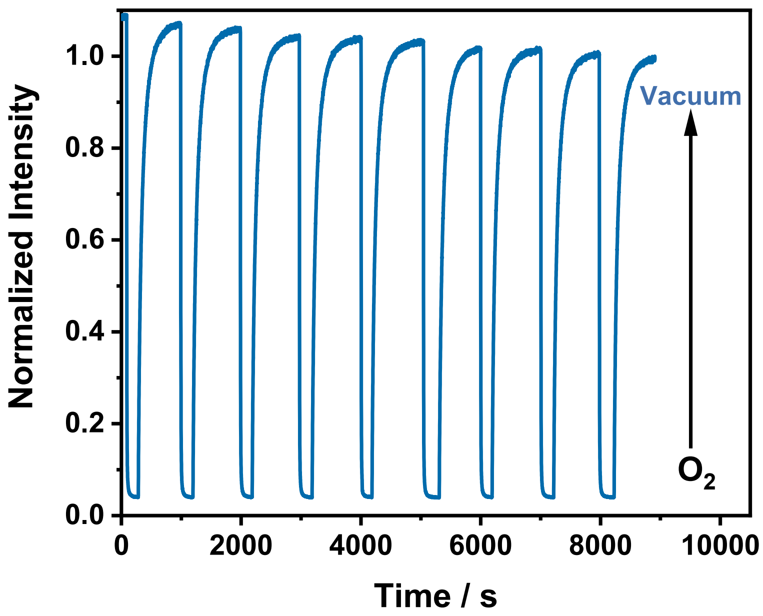

Figure S12. The time-dependent spectra of CuIBP when switching between vacuum and 1 bar O_2, excited by 365 nm UV light and detected at 542 nm.

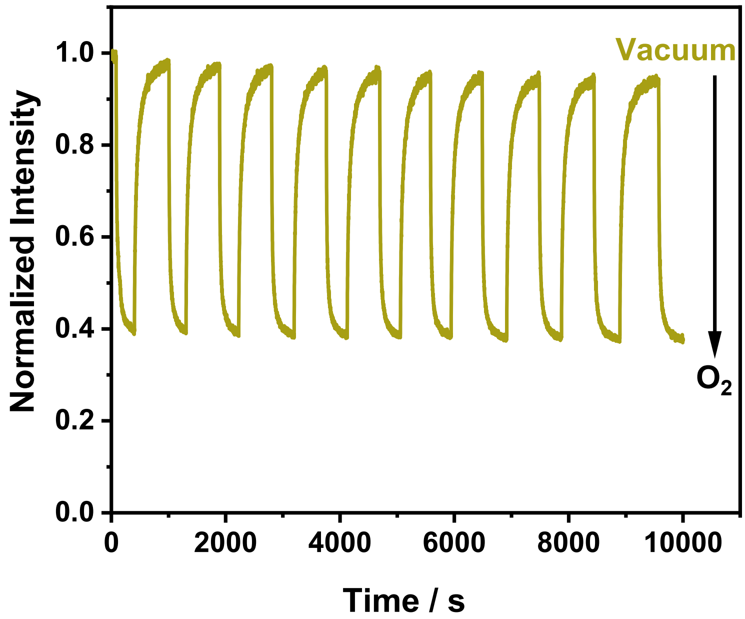

Figure S13. The time-dependent spectra of CuBrBP when switching between vacuum and 1 bar O_2, excited by 365 nm UV light and detected at 577 nm.

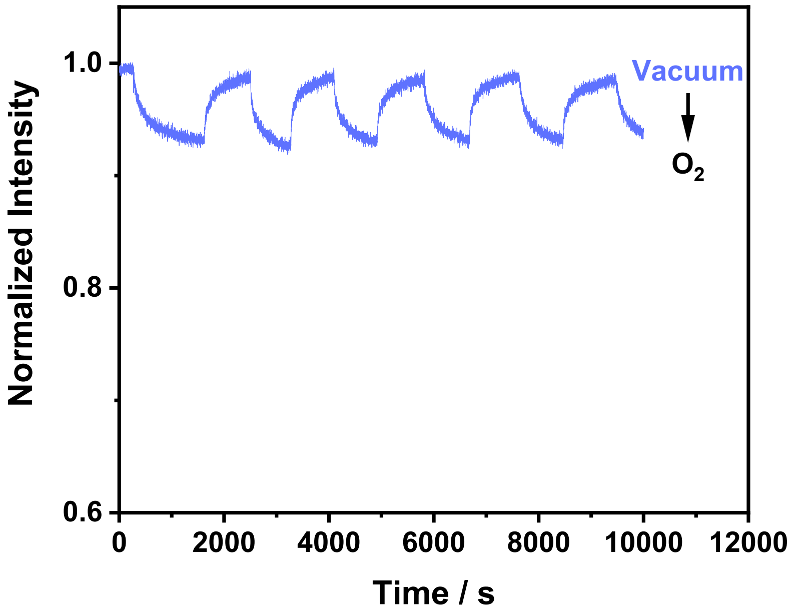

Figure S14. The time-dependent spectra of CuClBP when switching between vacuum and 1 bar O_2, excited by 365 nm UV light and detected at 605 nm.

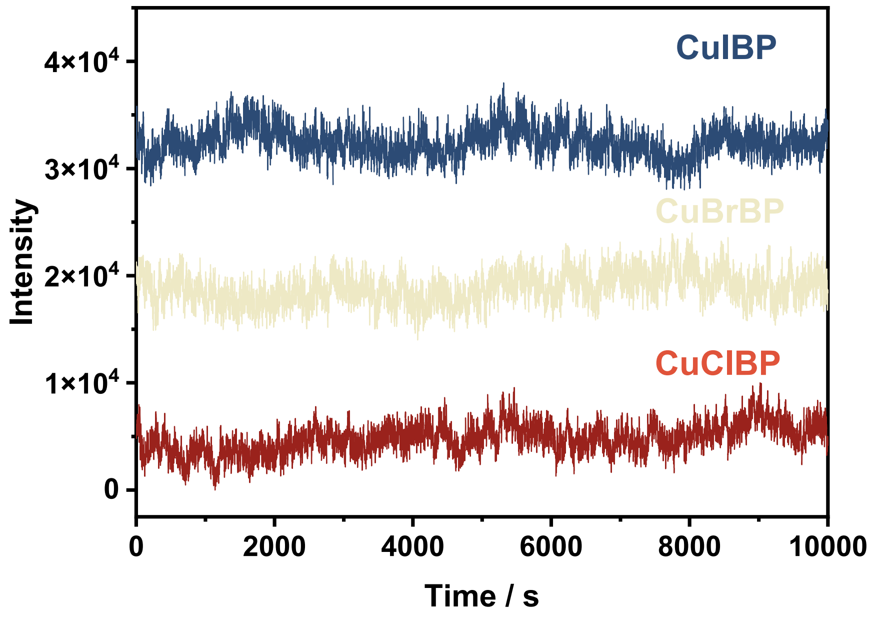

Figure S15. Photostability test of CuXBPs, excited by 365 nm UV light and detected at λ_{em}.

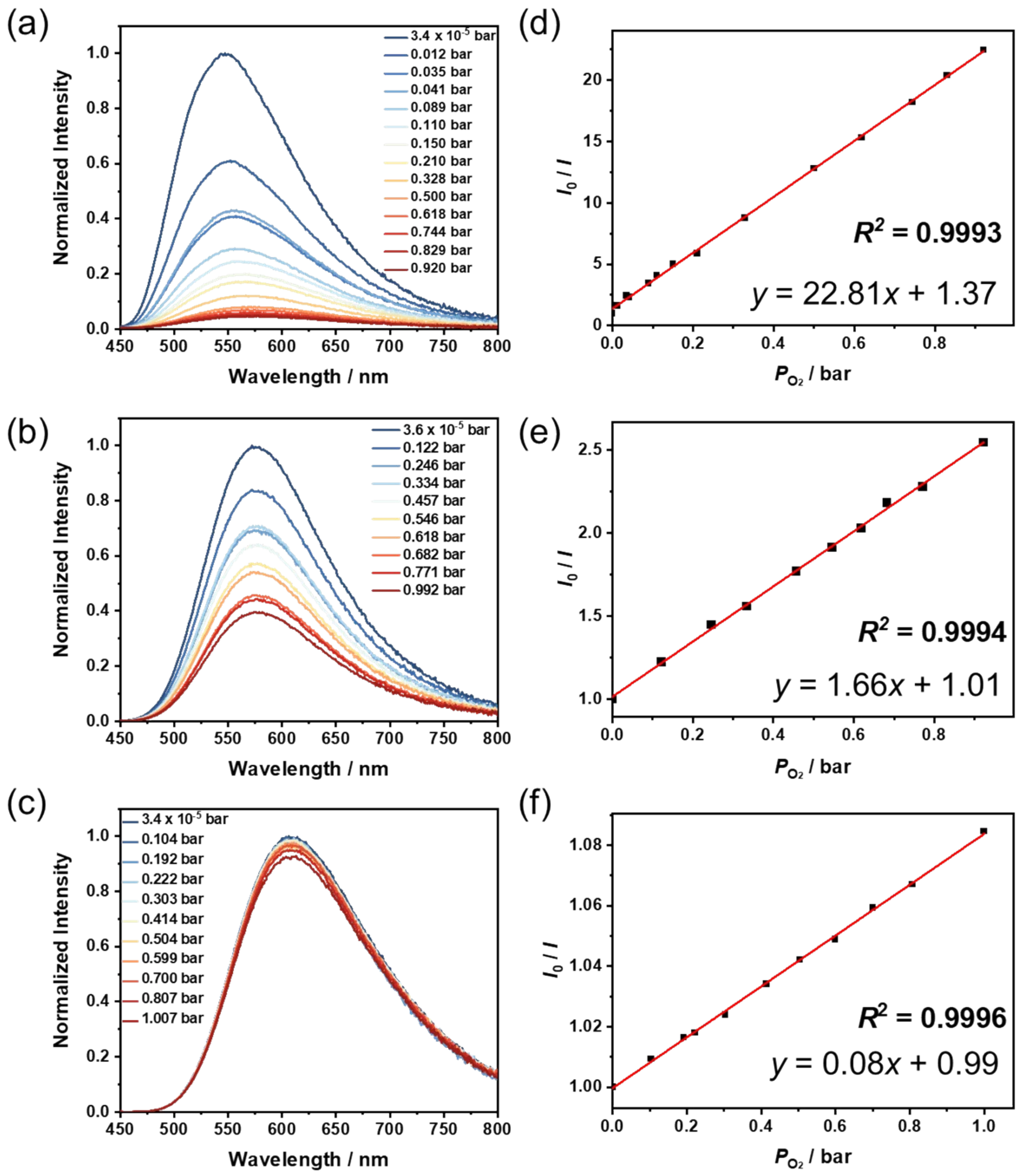

Figure S16. (a-c) Emission spectra of CuXBPs (X = I, Br, Cl) at different O_2 pressures, excited at 365 nm. (d-f) The Stern–Volmer plots of CuIBP, CuBrBP and CuClBP at different O_2 pressures, detected at λ_{em}.

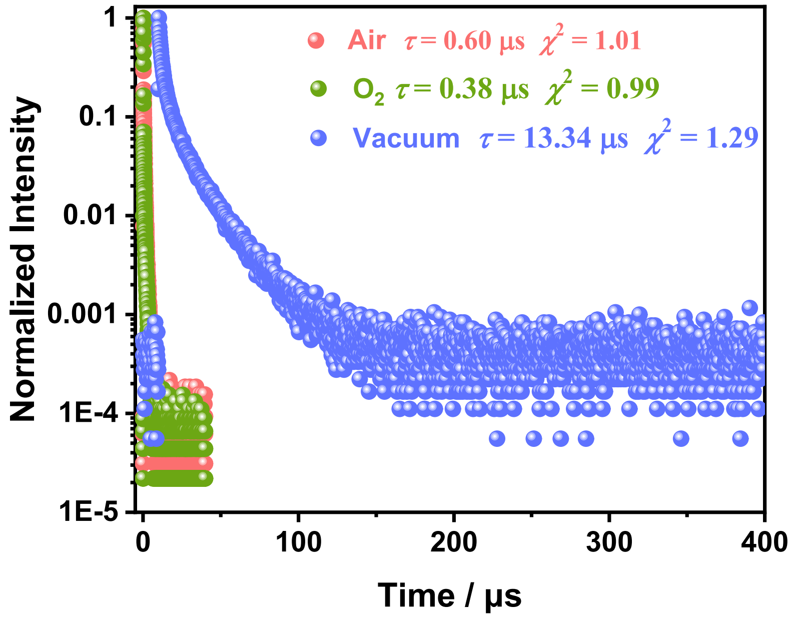

Figure S17. Luminescence decay curves of CuIBP in air, O_2 and vacuum, respectively, excited by 375-nm VPL and detected at 542 nm.

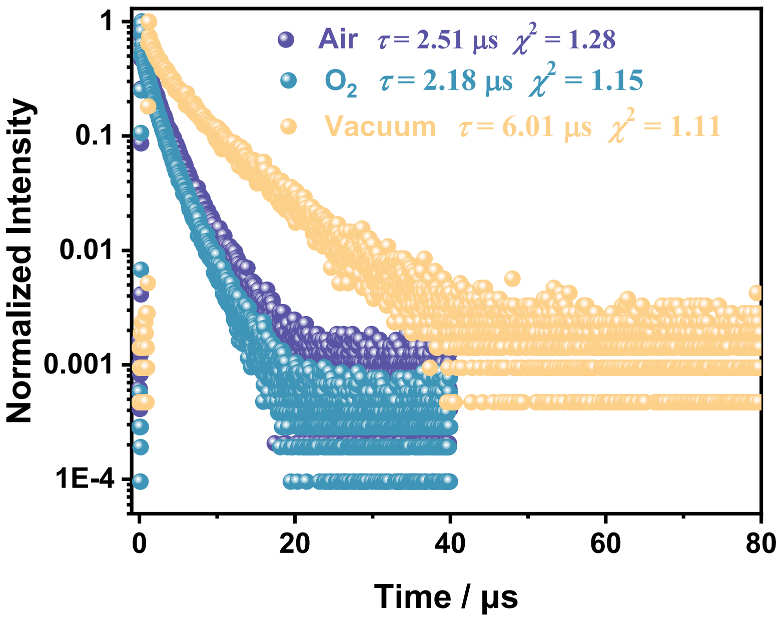

Figure S18. Luminescence decay curves of CuBrBP in air, O_2 and vacuum, respectively, excited by 375-nm VPL and detected at 577 nm.

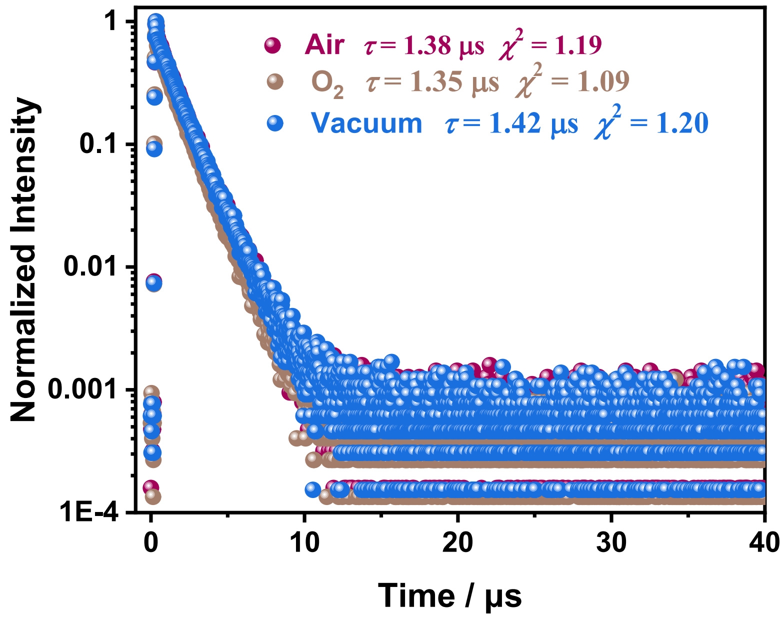

Figure S19. Luminescence decay curves of CuClBP in air, O_2 and vacuum, respectively, excited by 375-nm VPL and detected at 605 nm.

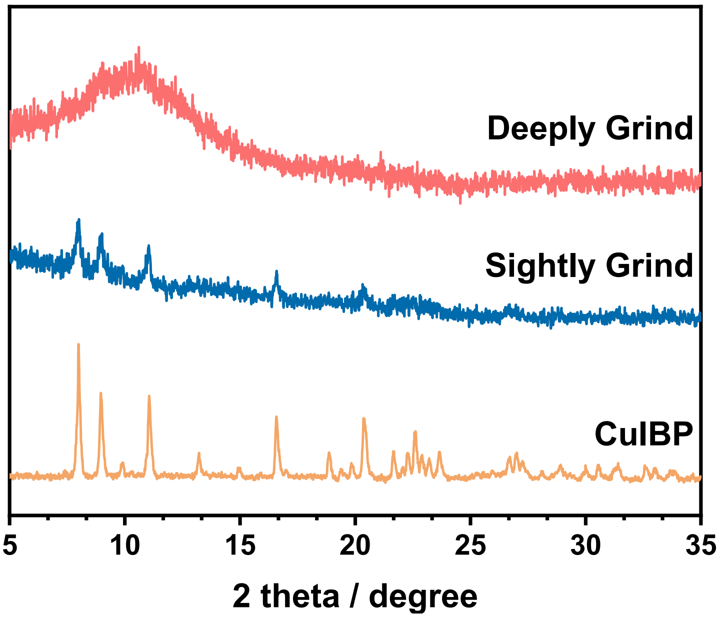

Figure S20. PXRD patterns of CuIBP after deeply and sightly grinding.

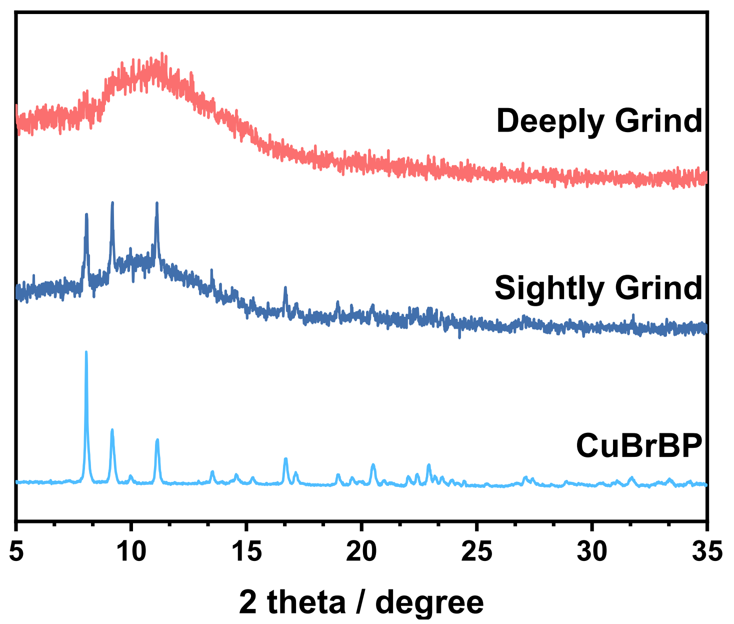

Figure S21. PXRD patterns of CuBrBP after deeply and sightly grinding.

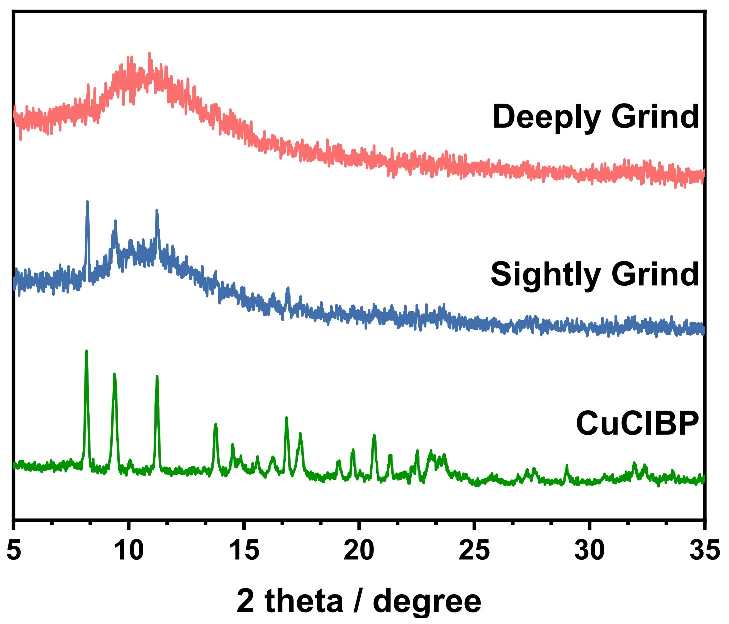

Figure S22. PXRD patterns of CuClBP after deeply and sightly grinding.

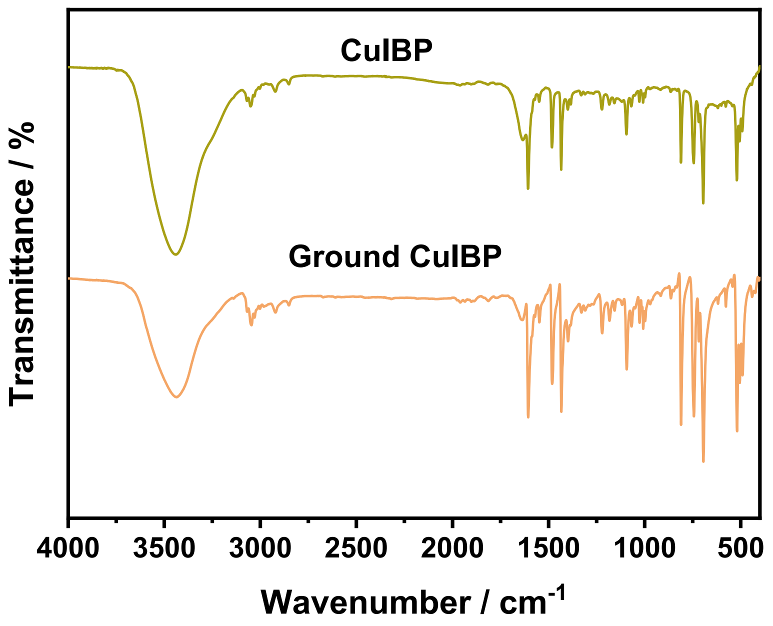

Figure S23. The FT-IR spectra of CuIBP before and after grinding.

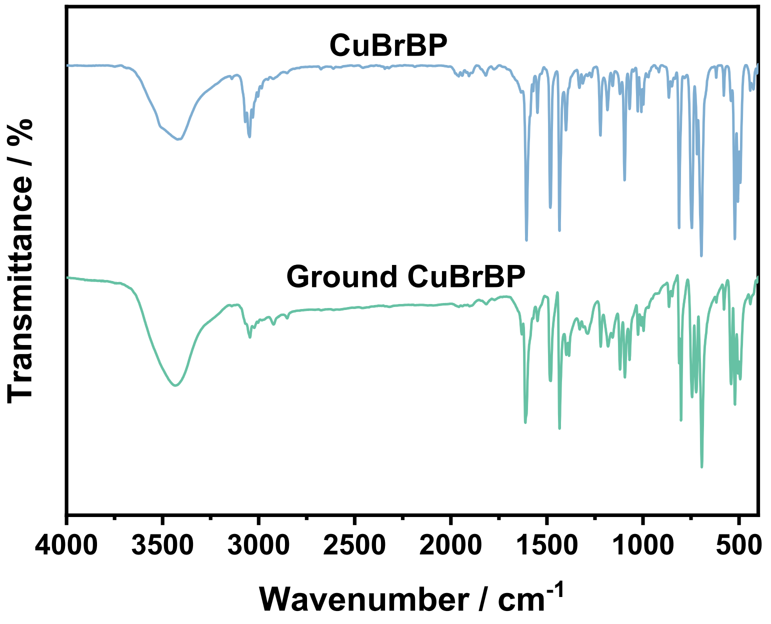

Figure S24. The FT-IR spectra of CuBrBP before and after grinding.

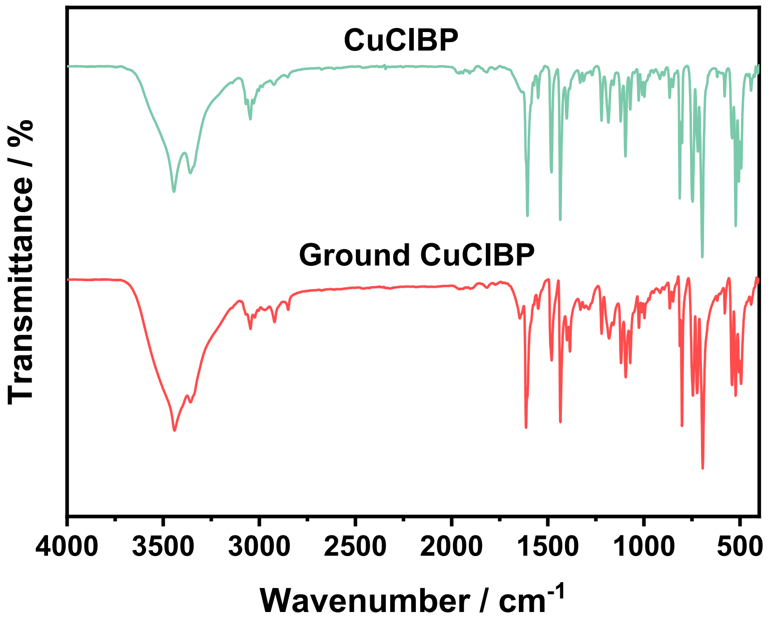

Figure S25. The FT-IR spectra of CuClBP before and after grinding.

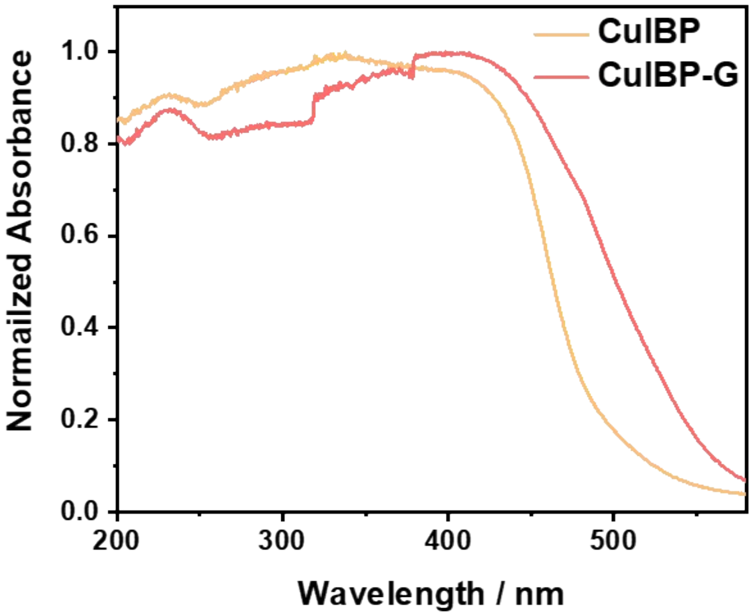

Figure S26. Solid-State UV-vis absorption spectra of CuIBP and CuIBP-G.

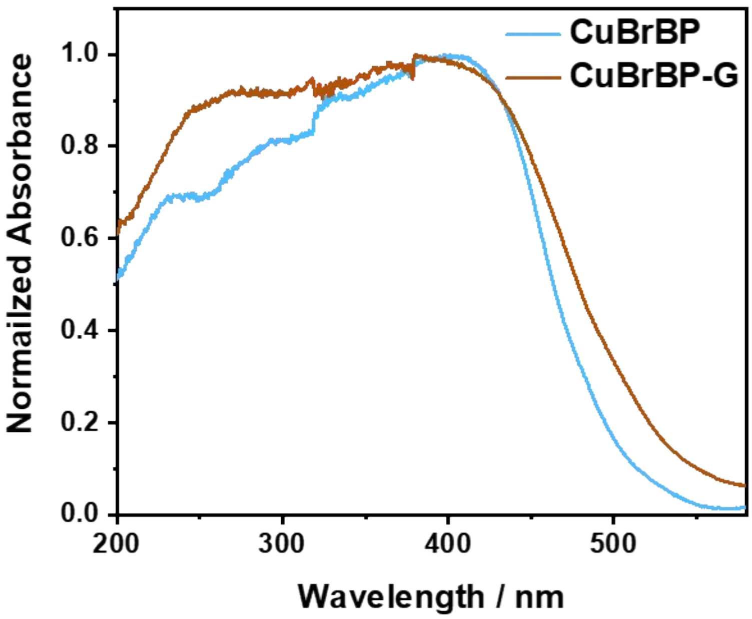

Figure S27. Solid-State UV-vis absorption spectra of CuBrBP and CuBrBP-G.

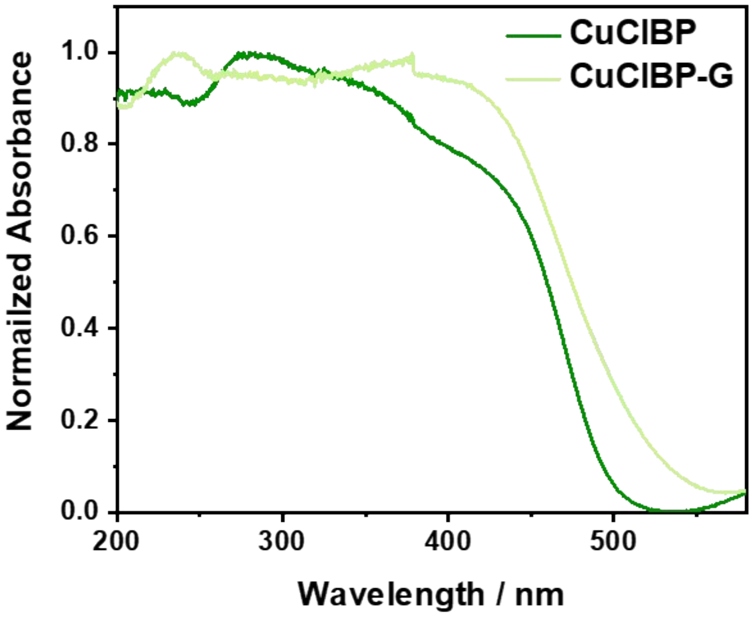

Figure S28. Solid-State UV-vis absorption spectra of CuClBP and CuClBP-G.

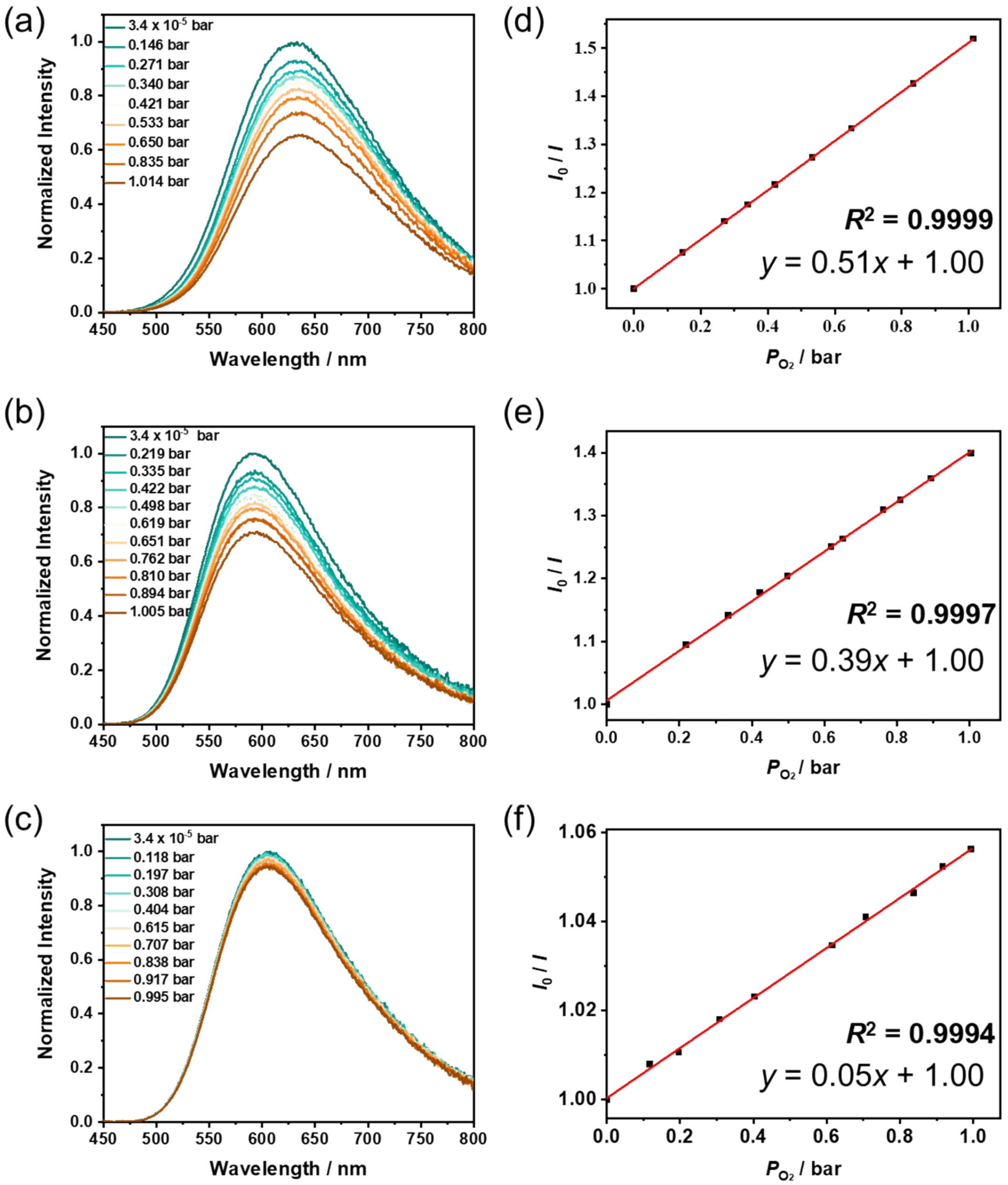

Figure S29. (a-c) Emission spectra of CuIBP-G, CuBrBP-G and CuClBP-G at different O_2 pressures, excited at 365 nm. (d-f) The Stern–Volmer plots of CuIBP-G, CuBrBP-G and CuClBP-G at different O_2 pressures, detected at λ_{em}.

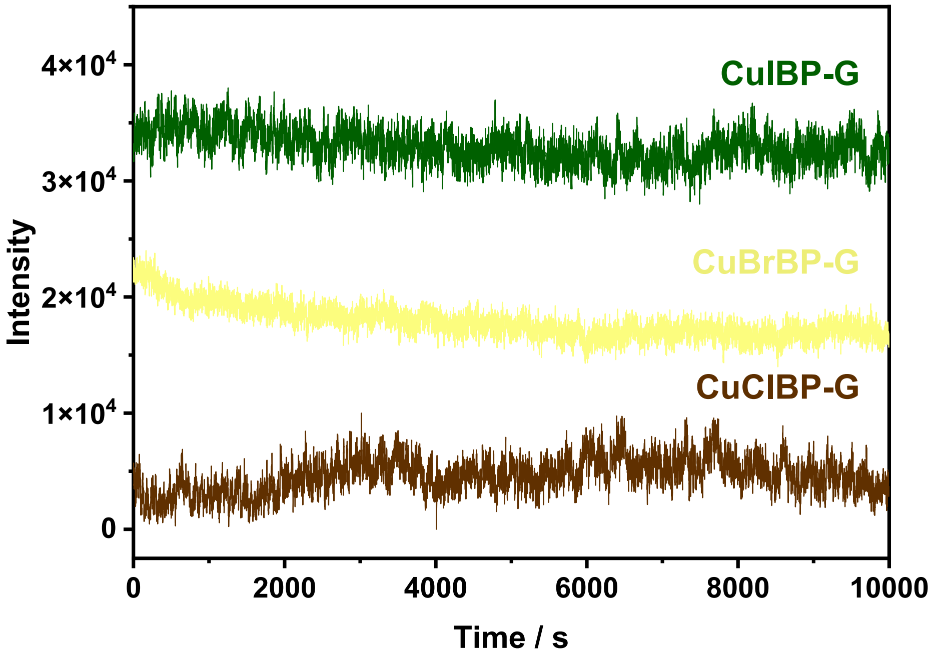

Figure S30. Photostability test of CuXBP-G, excited by 365 nm UV light and detected at λ_{em}.

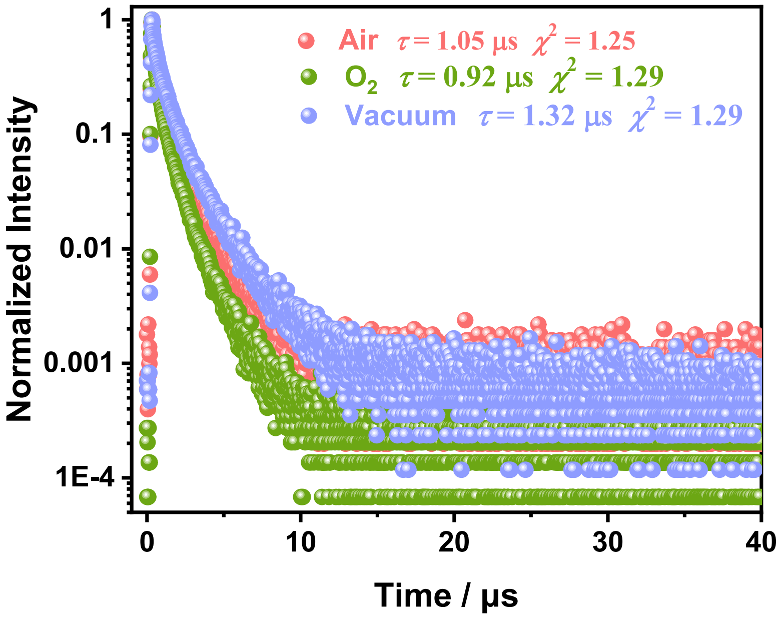

Figure S31. Luminescence decay curves of CuIBP-G in air, O_2 and vacuum, respectively, excited by 375-nm VPL and detected at 627 nm.

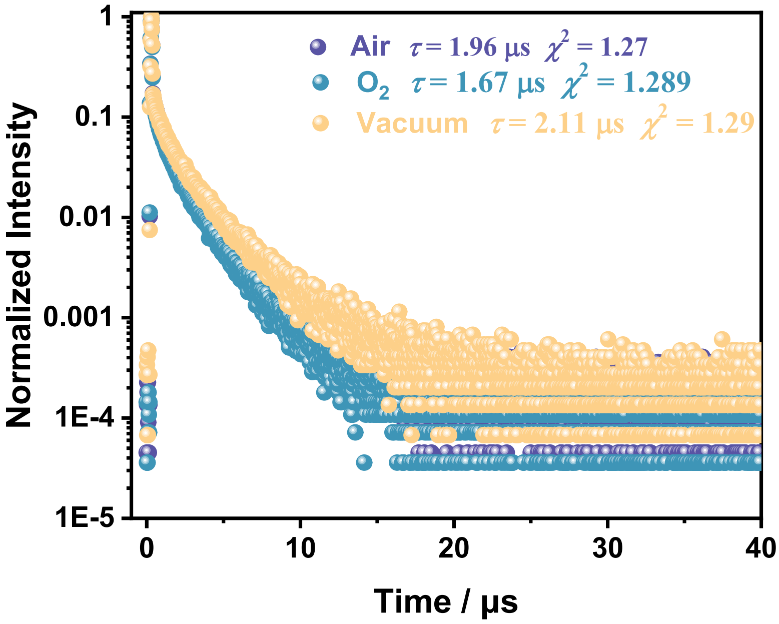

Figure S32. Luminescence decay curves of CuBrBP-G in air, O_2 and vacuum, respectively, excited by 375-nm VPL and detected at 593 nm.

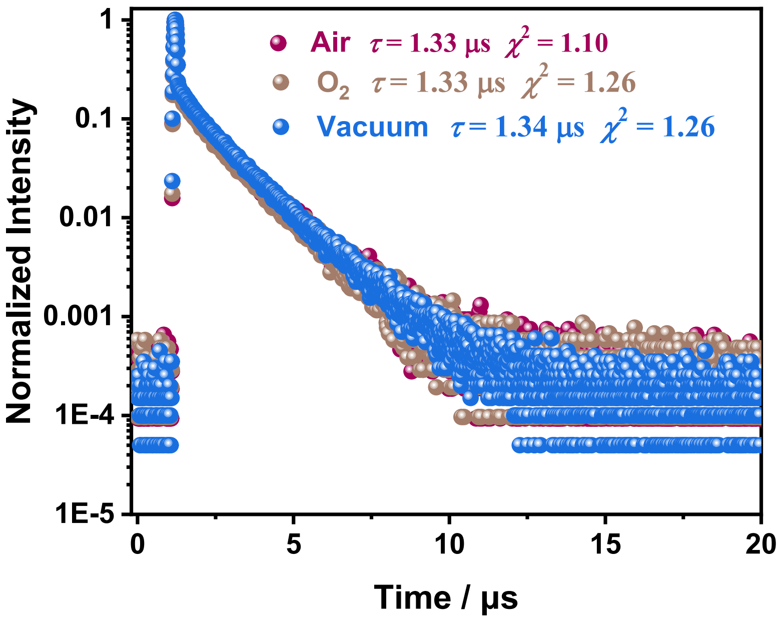

Figure S33. Luminescence decay curves of CuClBP-G in air, O_2 and vacuum, respectively, excited by 375-nm VPL and detected at 607 nm.

Table S1. Crystallographic data and structural refinement parameters of $CuXBP\cdot CH_3OH$.

Compounds	$CuIBP\cdot CH_3OH$	$CuBrBP\cdot CH_3OH$	$CuClBP\cdot CH_3OH$
CCDC No.	2473355	2473359	2473358
Formula	$C_{52.67}H_{44.67}Cu_2I_2N_2O_{0.67}P_2$	$C_{52.67}H_{44.67}Cu_2Br_2N_2O_{0.67}P_2$	$C_{52.67}H_{44.67}Cu_2Cl_2N_2O_{0.67}P_2$
Formula weight	1159.48	1065.17	976.25
T / K	297.12(10)	294.5(3)	296.9(2)
Crystal system	triclinic	triclinic	triclinic
Space group	P-1	P-1	P-1
a / Å	9.3425(1)	9.2653(3)	9.2424(1)
b / Å	12.0825(2)	12.0008(4)	11.8969(2)
c / Å	12.1874(2)	12.0094(4)	11.9954(2)
α / °	109.651(1)	109.053(3)	108.728(1)
β / °	99.347(1)	100.228(3)	100.850(1)
γ / °	100.868(1)	100.638(3)	100.625(1)
Volume / Å^3	1233.85(3)	1199.15(7)	1183.54(3)
Z	1	1	1
R_{int}	0.0499	0.0302	0.0247
R_1 $[I > 2\sigma(I)]^a$	0.0307	0.0312	0.0356
wR_2 $[I > 2\sigma(I)]^b$	0.0831	0.0929	0.1099
R_1 (all data)	0.0307	0.0312	0.0356
wR_2 (all data)	0.0840	0.0961	0.1128
GOF	1.073	1.035	1.086

$^a R_1 = \Sigma||F_o| - |F_c|| / \Sigma |F_o|$

$^b wR_2 = [\Sigma w(F_o^2 - F_c^2)^2/\Sigma w(F_o^2)^2]^{1/2}$

Table S2. Crystallographic data and structural refinement parameters of CuXBP.

Compounds	CuIBP	CuBrBP	CuClBP
CCDC No.	2473360	2473356	2473357
Formula	$C_{52}H_{42}Cu_2I_2N_2P_2$	$C_{52}H_{42}Cu_2Br_2N_2P_2$	$C_{52}H_{42}Cu_2Cl_2N_2P_2$
Formula weight	1137.69	1043.71	954.79
T / K	293.8(2)	296.93(10)	297.38(10)
Crystal system	triclinic	triclinic	triclinic
Space group	P-1	P-1	P-1
a / Å	9.3779(1)	9.2949(3)	9.2912(2)
b / Å	12.0370(2)	11.8875(5)	11.7601(2)
c / Å	12.1052(2)	11.9565(4)	11.7999(2)
α / °	108.854(2)	108.230(3)	107.136(2)
β / °	99.325(1)	100.230(3)	100.739(1)
γ / °	101.378(1)	100.811(3)	100.697(2)
Volume / Å^3	1229.61(3)	1192.35(8)	1170.11(4)
Density / g·cm^{-1}	1.536	1.454	1.355
Z	1	1	1
R_{int}	0.0324	0.0307	0.0218
R_1 [$I > 2\sigma(I)$] [a]	0.0218	0.0330	0.0324
wR_2 [$I > 2\sigma(I)$] [b]	0.0499	0.0955	0.0992
R_1 (all data)	0.0218	0.0330	0.0324
wR_2 (all data)	0.0508	0.0973	0.1003
GOF	1.040	1.052	1.059

[a] $R_1 = \Sigma||F_o| - |F_c|| / \Sigma |F_o|$

[b] $wR_2 = [\Sigma w(F_o^2 - F_c^2)^2/\Sigma w(F_o^2)^2]^{1/2}$

Table S3 The main bond lengths of CuXBP.

Compounds	CuIBP	CuBrBP	CuClBP
Cu-Cu / Å	3.18	3.08	3.03
Cu-X / Å	2.66	2.58	2.38
Cu-P / Å	2.23	2.21	2.19
Cu-N / Å	2.07	2.07	2.06

Table S4. Elemental analyses of CuXBP (X = I, Br, Cl).

Compounds	Calculated Result			Experimental Result		
	C / %	N / %	H / %	C / %	N / %	H / %
CuIBP	54.89	2.46	3.72	54.67	2.51	3.55
CuBrBP	59.84	2.68	4.06	59.55	2.58	4.29
CuClBP	65.41	2.93	4.43	65.01	3.08	5.08

Table S5. The orbital occupancy of the $S_0 \rightarrow S_1$ transition in CuXBP (X = I, Br, Cl) was calculated by analysing the Mulliken orbital composition.

Compounds	S_0		S_1		Difference of S_0 and S_1		XLCT/MLCT ratio
	Halogen / %	Cu / %	Halogen / %	Cu / %	Halogen / %	Cu / %	
CuIBP	44.97	35.46	0.16	0.88	44.81	34.58	1.30
CuBrBP	34.57	44.51	0.19	0.97	34.38	43.54	0.79
CuClBP	25.32	52.46	0.14	0.99	25.18	51.47	0.49

Table S6. The occupancy of the $S_0 \rightarrow T_1$ transition in CuXBP (X = I, Br, Cl) was calculated by analysing the Mulliken orbital composition.

Compounds	S_0		T_1		Difference of S_0 and T_1		XLCT/MLCT ratio
	Halogen / %	Cu / %	Halogen / %	Cu / %	Halogen / %	Cu / %	
CuIBP	25.07	33.31	0.36	1.96	24.71	31.35	0.79
CuBrBP	31.01	41.98	0.43	1.85	30.58	40.13	0.76
CuClBP	22.22	49.58	0.37	2.00	21.85	47.58	0.46

Reference

[1] M. J. Frisch, G. W. Trucks, H. B. Schlegel, G. E. Scuseria, M. A. Robb, J. R. Cheeseman, G. Scalmani, V. Barone, B. Mennucci, G. A. Petersson, H. Nakatsuji, M. Caricato, X. Li, H. P. Hratchian, A. F. Izmaylov, J. Bloino, G. Zheng, J. L. Sonnenberg, M. Hada, M. Ehara, K. Toyota, R. Fukuda, J. Hasegawa, M. Ishida, T. Nakajima, Y. Honda, O. Kitao, H. Nakai, T. Vreven, Jr J. A. Montgomery, J. E. Peralta, F. Ogliaro, M. Bearpark, J. J. Heyd, E. Brothers, K. N. Kudin, V. N. Staroverov, R. Kobayashi, J. Normand, K. Raghavachari, A. Rendell, J. C. Burant, S. S. Iyengar, J. Tomasi, M. Cossi, N. Rega, J. M. Millam, M. Klene, J. E. Knox, J. B. Cross, V. Bakken, C. Adamo, J. Jaramillo, R. Gomperts, R. E. Stratmann, O. Yazyev, A. J. Austin, R. Cammi, C. Pomelli, J. W. Ochterski, R. L. Martin, K. Morokuma, V. G. Zakrzewski, G. A. Voth, P. Salvador, J. J. Dannenberg, S. Dapprich, A. D. Daniels, O. Farkas, J. B. Foresman, J. V. Ortiz, J. Cioslowski, D. J. Fox. *Gaussian, Inc.*, Wallingford CT, **2009**.

[2] C. Adamo, V. Barone. Toward reliable density functional methods without adjustable parameters: The PBE0 model. *J. Chem. Phys.* **1999**, 110, 6158.

[3] T. Lu, F. Chen. *J. Comput. Chem*. **2012**, 33, 580.
